# An Efficient Machine Learning Model Based on Improved Features Selections for Early and Accurate Heart Disease Predication

**DOI:** 10.1155/2022/1906466

**Published:** 2022-07-13

**Authors:** Farhat Ullah, Xin Chen, Khairan Rajab, Mana Saleh Al Reshan, Asadullah Shaikh, Muhammad Abul Hassan, Muhammad Rizwan, Monika Davidekova

**Affiliations:** ^1^School of Automation, China University of Geosciences, Wuhan 430074, China; ^2^College of Computer Science and Information Systems Najran University, Najra 61441, Saudi Arabia; ^3^Department of Computing and Technology, Abasyn University Peshawar, Peshawar 25000, Pakistan; ^4^Secure Cyber Systems Research Group, WMG, University of Warwick, Coventry CV4 7AL, UK; ^5^Information Systems Department, Faculty of Management Comenius University in Bratislava Odbojárov 10, Bratislava 82005 25, Slovakia

## Abstract

Coronary heart disease has an intense impact on human life. Medical history-based diagnosis of heart disease has been practiced but deemed unreliable. Machine learning algorithms are more reliable and efficient in classifying, e.g., with or without cardiac disease. Heart disease detection must be precise and accurate to prevent human loss. However, previous research studies have several shortcomings, for example,take enough time to compute while other techniques are quick but not accurate. This research study is conducted to address the existing problem and to construct an accurate machine learning model for predicting heart disease. Our model is evaluated based on five feature selection algorithms and performance assessment matrix such as accuracy, precision, recall, F1-score, MCC, and time complexity parameters. The proposed work has been tested on all of the dataset'sfeatures as well as a subset of them. The reduction of features has an impact on theperformance of classifiers in terms of the evaluation matrix and execution time. Experimental results of the support vector machine, K-nearest neighbor, and logistic regression are 97.5%,95 %, and 93% (accuracy) with reduced computation timesof 4.4, 7.3, and 8seconds respectively.

## 1. Introduction

Chronic heart diseases are one of the most dangerous and life-threatening worldwide. The fundamental cause of heart failure is narrowing and blockage of coronary arteries, where the heart fails to supply enough blood to other organs [1, 2]. The coronary arteries must be accessible to supply blood to the heart. According to a recent study, heart disease is the most common disease in the United States and worldwide with a high percentage of heart disease patients [3]. Common symptoms are shortness of breath, swelling feet, and tiredness [4]. Junk food with a maximum number of cholesterols, smoking, poor nutrition, high blood pressure, and physical inactivity increase the risks of heart disease [5]. Heartburn, stroke, and heart attack are all symptoms of coronary artery disease (CAD). Other heart disorders include heart rhythm problems, congenital heart disease, congestive heart failure, and cardiovascular disease. Traditional methods for detecting cardiac disease were used [6]. Lack of medical understanding and diagnostic instruments, on time detecting, and treating heart disease in poor countries is very difficult [7, 8]. The main motivation behind the research study is to propose a comprehensive and precise diagnosis technique for heart disease to avoid loss of lives. Cardiovascular disease is the leading cause of death in both developed and developing countries. According to the WHO, 17.90 million people died from cardiovascular disease (CVD) in 2016, accounting for 30% of all deaths globally. Moreover, 0.2 million Pakistanis per year face death and death counts are still uplifting per year. According to the European Society of Cardiology (ESC), there are 26.5 million people in Europe who suffer from heart disease, with 3.8 million new cases being discovered each year. Heart disease kills 50–55% of patients in the first year, and treatment costs 4% of the yearly healthcare expenditure [9]. Invasive diagnostic procedures relied on a patient's medical history, physical examination results, and an examination of symptoms to make a diagnosis of heart disease [10]. Traditional methods like angiography are regarded as the most precise practice when it comes to detecting heart abnormalities but still facing certain limitations, such as high costs, various other side effects, and a high level of technical expertise is required, and most importantly it is much expensive, computationally difficult, and take time to assess [11, 12], to overcome the limitations of conventional invasive-based approaches for detecting cardiac disease. Predictive machine learning and deep learning algorithms were used to construct noninvasive Internet of Medical Thing (IoMT) [13–16], smart healthcare systems such as KNN, SVM, NB, DT, LR, RF, and ANN [17–22]. As a result, the death rate among individuals with heart disease has exponentially dropped per year.

The main objectives of this research study are as follows:

- To develop an intelligent medical decision system for the identification of cardiac illness on time.
- Machine learning classification methods such as decision tree (DT), stochastic gradient descent (SGD), K-nearest neighbor (KNN), naive Bayes (NB), random forest (RF), logistics regression (LR), and support vector machine (SVM) are used to select the best model for early heart disease diagnosis.
- Feature selection such as LASSO, ANOVA, MultiSURF, variance threshold, and mutual information to identify the most important and linked features that properly reflect the pattern of the desired target.
- Cleveland hospital datasets related to heart disease are utilized.

The rest of the paper is organized as follows: Section 2 provides an overall literature review, materials and methods are explained in Section 3, results and discussion are discussed in Section 4, and Section 5 provides a conclusion.

## 2. Literature Review

Over time experts and practitioners have shown keen interest in diagnosing heart disease by employing classical machine learning techniques. Experts usually utilize a classification approach to create a heart disease diagnosis model in their research study [5, 23–38]. The machine learning model can diagnose heart failure with 99% accuracy, according to preliminary computational results as shown in Table 1.

Current research has imbalanced distribution, e.g., some approaches are accurate but required a long time for computation, and some techniques responded on time but are not very accurate to diagnose such serious disease. As a result, there is a great deal of work to improve the performance evaluation rate in this area.

## 3. Materials and Methods

**Figure 1:**
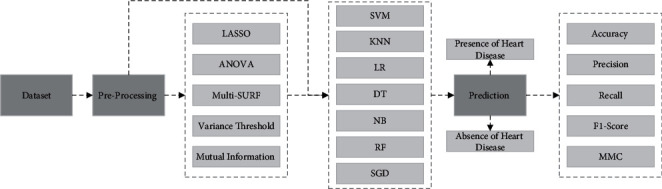
Proposed methodology to predict heart disease.

The suggested approach aims to distinguish patients with or without cardiac disease. Both complete and selective features are enforced to investigate predictive models. Important features are identified using methods, e.g., LASSO, ANOVA, MultiSURF, variance threshold, and mutual information. K-nearest neighbor (KNN), support vector machine (SVM), decision tree (DT), random forest (RF), logistic regression (LR), stochastic gradient descent (SGD), and naive Bayes (NB) machine learning algorithms are deployed in the system for classification. Structure based on four steps, including exploratory data analysis, feature selection, ML classifiers, and performance evaluation matrix approach, is adopted. Algorithm 1 and Figure 1depict the proposed system's framework.

### 3.1. Preprocessing

Cleaning data is very important to achieve maximum accuracy and actual efficiency of machine learning algorithms. Different data preparation techniques are used to ensure each and every features must have the same coefficient. Moreover, standard scalar assures that each feature has the same mean, while min-max scalar shifts of data are set between 0 and 1, and lastly the row with missing values is erased.

### 3.2. Feature Selection

Precise and accurate feature selection is a very important parameter because it improves classification accuracy with minimum time complexity. LASSO, ANOVA, MultiSURF, variance threshold, and mutual information feature selection algorithms are used to select features from the dataset.

In the LASSO algorithm, some coefficients (feature) become zero, and are removed from the feature subset, derived from equations (1)–(6), while ANOVA compares the mean of two or more groups that are statistically distinct, derived from equations (7)–(11). MultiSURF is the most reliable feature selection algorithm explained in equations (12) and (13) and can be used for explicitly detecting pure 2-way interactions across a wide range of problems. Variance threshold is efficient in eliminating all features with variance below a certain threshold evaluated from equation (20). Lastly, we used mutual information in the feature selection phase to find dimensionless quantities with units of bits that measure “how much one random variable provides information about another.” Mathematical modulation behind mutual information is explained in equation (15)–(20).

We have *N* number of samples {(*xᵢ*, *yᵢ*)} *ᴺᵢ*₌₁ in the linear regression, where each *xᵢ* = (*xᵢ₁*,…,*xᵢp*) is a p-dimensional vector of features, and each *yᵢ* ∈ ℝ is the corresponding response variable. Our goal is to use a linear mixture of features to approximate the response variable yᵢ. Then the cost function (or loss function) must be optimized by using MSE as a cost function to determine the best fit line.

(1) $$\begin{array}{c} \mathrm{LASSO}=\eta \left(x_{i}\right)=\beta_{0}+\sum_{5=1}^{P}x_{\text{ij}}\beta_{j}, \end{array}$$

(2) argmin β0,β1N∑i=1Nyi−β0−∑j=1pxijβj^2.

The following equation shows the closed form solution that determines the coefficients of the aforesaid cost function. LASSO reduces the coefficients of redundant variables to zero, allowing the direct feature method. The LASSO cost function is as follows:

(3) $$\begin{array}{c} \beta ={\left(X^{T}X\right)}^{-1}X^{T}Y, \end{array}$$

(4) argminβ0,β1N∑i=1Nyi−β0−∑j=1pxijβj^2λ∑j=1pβj,

(5) $$\begin{array}{c} E\left(\beta \right)+\lambda R\left(\beta \right). \end{array}$$

In equation (6) argmin finds values where the expression *E*(*β*) + *R*(*β*) is minimum. The sparsity (*β* ^*∗*^) of a model is defined by the number of parameters in *β* ^*∗*^ that are exactly equal to zero. In real-world problems, we need the model to take up only the most useful traits. LASSO regularization yields sparse solutions, which automatically choose features.

(6) $$\begin{array}{c} \beta^{*}=\arg\min\left[E\left(\beta \right)+\lambda R\left(\beta \right)\right.. \end{array}$$

ANOVA makes use of the more traditional, standardized nomenclature. When we look at equations, we can see that the divisor has a degree of freedom (DF), the total is sum of squares (SS), we get mean square (MS), and the squared terms represent deviations from the sample mean. As a starting point, SS is partitioned into components that correspond to the model's effects.

(7) $$\begin{array}{c} \text{ANOVA}={\text{Ms}}^{2}=\frac{1}{n-1}\sum_{i}{\left(y_{i}-\bar{y}\right)}^{2}. \end{array}$$

(8) $$\begin{array}{c} {\text{ss}}_{\text{Total}}={\text{ss}}_{\text{Error}}+{\text{ss}}_{\text{Treatments}}. \end{array}$$

Similarly, the number of degrees of freedom (DF) can be partitioned: one of these components specifies chi-squared distribution for error that represents the related sum of squares, and the same “treatments” have no effect if there is no value.

(9) $$\begin{array}{c} \text{DF}={\text{DF}}_{\text{Error}}+{\text{DF}}_{\text{Treatments}}. \end{array}$$

In lieu of the more traditional one-way analysis of ANOVA, the following form can be used to express each piece of information.

(10) $$\begin{array}{c} y_{\text{ij}}=\mu +T_{j}+\varepsilon_{\text{ij}}, \end{array}$$

(11) $$\begin{array}{c} \sum_{J=1}^{C}T_{j}=0. \end{array}$$

In the case of the Multi-SURF algorithm, each feature in the dataset is assigned to one of two groups. Inside the data collection, each feature should be scaled 0–1 and repeat the process *m* times with a p-long weight vector (W) of zeros. Then the feature vector (*X*) of a random instance and the feature vectors of the instances closest to *X* by Euclidean distance. It refers to the closest same-class instance, whereas it refers to the nearest different-class instance. In equation (13) we compute a two-tailed *p*-value using the cumulative distribution function to determine the number of cases that are close or distant.

(12) $$\begin{array}{c} \text{MultiSURF}=w_{i}=w_{i}-{\left(x_{i}-{\text{nearHit}}_{i}\right)}^{2}+{\left(x_{i}-{\text{nearMiss}}_{i}\right)}^{2}, \end{array}$$

(13) $$\begin{array}{c} 2\left(1-\frac{1}{\sqrt{2\pi }}\int_{-\infty }^{\left(1/2\right)}\text{e}\frac{-x}{2}\text{d}x\right)\approx 0.60. \end{array}$$

The information-theoretic formula is used by the variance threshold algorithm to reduce dataset features. For a given feature subset *Q*, there are a variety of truth value assignments. A feature set *Q* divides training data into groups of instances with the same truth value into a set of training data instances. The entropy of positive *P*_*i*_ and negative *n*_*i*_ class values are calculated by using the below equation.

(14) $$\begin{array}{c} \text{Variance Threshold}\left(Q\right)=-\sum_{1=0}^{2\left|Q\right|-1}\frac{P_{i}+n_{i}}{\left|\text{Sample}\right|}\left[\frac{P_{i}}{P_{i}+N_{i}}{\text{log}}_{2}\frac{P_{i}}{P_{i}+N_{i}}+\frac{n_{i}}{P_{i}+n_{i}}{\text{log}}_{2}\frac{n_{i}}{P_{i}+n_{i}}\right]. \end{array}$$

Mutual information, as opposed to correlation coefficients, includes information on all linear and nonlinear dependencies. However, if the joint distribution of *X* and Y is bivariate normal and both marginal distributions are normally distributed, the relationship between I and *p* is precise.

(15) $$\begin{array}{c} \text{Mutual Information}=H\left(X_{i}\right)=\frac{1}{2}\text{log}\left[2\pi \text{e}\sigma_{1}^{2}\right), \end{array}$$

(16) $$\begin{array}{c} \frac{1}{2}+\frac{1}{2}\text{log}\left(2\pi \right)+\text{log}\left(\sigma_{1}\right)i\epsilon \left\{1,2\right\}, \end{array}$$

(17) $$\begin{array}{c} H\left(x_{1}x_{2}\right)=\frac{1}{2}\text{log}{\left(2\pi e\right)}^{2}\left|\Sigma \right|, \end{array}$$

(18) $$\begin{array}{c} 1+\text{log}\left(2\pi \right)+\text{log}\left(\sigma_{1}\sigma_{2}\right)+\frac{1}{2}\text{log}\left(1-p^{2}\right), \end{array}$$

(19) $$\begin{array}{c} I\left(X_{1},X_{2}\right)=H\left(X_{1}\right)+\left(X_{2}\right)-H\left(X_{1},X_{2}\right), \end{array}$$

(20) $$\begin{array}{c} I=-\frac{1}{2}\text{Log}{\left(1-P\right)}^{2}. \end{array}$$

### 3.3. Classification

Heart patients and healthy patients are separated into groups using machine learning classification methods. In this phase, we will take a look at a few prominent classification approaches as well as the theoretical basis of those methods.

#### 3.3.1. Support Vector Machine (SVM)

SVM is an ML classification technique; this has mainly been used to solve classification issues. It uses a maximum margin strategy to solve a complex quadratic problem, and is employed in a variety of applications due to its high classification performance. Moreover, SVM is best suited for identifying the best hyperplane to separate the data, as shown in equations (21)–(23).

(21) $$\begin{array}{c} w.x+b=0, \end{array}$$

(22) $$\begin{array}{c} X_{1}*w+b\leq 1,\text{yi}=-1. \end{array}$$

(23) $$\begin{array}{c} X_{1}*w+b\geq 1,\text{yi}=+1. \end{array}$$

#### 3.3.2. Naïve Bayes (NB)

The NB method uses the conditional probability theorem as can be seen in equation (24), to classify new feature vectors and also find their conditional probability values. The conditionality likelihood of each vector is used to calculate the new vector class and is usually utilized for text-related problem classification.

(24) $$\begin{array}{c} p\left(x|y\right)=\frac{p\left(y\;|\;x\right)p\left(x\right)}{P\left(y\right)}. \end{array}$$

#### 3.3.3. Decision Tree (DT)

DT is also an ML approach where each node is a leaf node with internal and external nodes connected. The internal nodes make decisions and send child nodes to the next node, whereas the leaf node has no child nodes, and is labeled derived from the following equations:

(25) $$\begin{array}{c} I=-\sum_{C}P\left(C\right){\text{log}}_{2}p\left(c\right), \end{array}$$

(26) $$\begin{array}{c} \text{Gain}\left(A\right)=I-I\left(\text{res}\right), \end{array}$$

(27) $$\begin{array}{c} I=1-\sum_{j}p{\left(c\right)}^{2}, \end{array}$$

(28) $$\begin{array}{c} G=1-\sum \frac{cj}{c}I\left(c\right), \end{array}$$

(29) $$\begin{array}{c} d=\sum_{i=1}^{k}|{\left(x-yi\right)}^{2}. \end{array}$$

#### 3.3.4. K-Nearest Neighbor (KNN)

KNN uses the similarity of new input to the incoming input samples in the training set and to predict a new input's class label, as shown in the following equation:

(30) $$\begin{array}{c} d=\sum_{i=1}^{k}|{\left(x-yi\right)}^{2}. \end{array}$$

#### 3.3.5. Logistic Regression (LR)

Binary classification problems are solved using a logistic regression technique, which predicts values for variables 0 and 1, and classifies them into two groups: negative (0) or positive (1). A threshold value of 0.5 is used in the multi-classification approach to predict decimal numbers, which is then used to classify the two classes, e.g., 0 and 1. Hypothesis if threshold ≥0.5 predicts 1, indicating that the patient has heart disease (cardiomyopathy). The mathematical representation of logistic regression is explained in the following equations:

(31) $$\begin{array}{c} P\left(x\right)=\frac{1}{1+{\text{e}}^{-\left(\left(x-\mu \right)/s\right)}}, \end{array}$$

(32) $$\begin{array}{c} P\left(x\right)=\frac{1}{1+{\text{e}}^{-\left(\beta 0-\beta 1x\right)}}, \end{array}$$

(33) $$\begin{array}{c} -\text{yk In }pk-\left(1-\text{yk}\right)\text{In}\left(1-pk\right), \end{array}$$

(34) $$\begin{array}{c} \text{e}=\sum_{k:\text{yk}=1}\text{In}\left(\text{pk}\right)+\sum_{k:\text{yk}=0}\text{In}\left(1-\text{pk}\right), \end{array}$$

(35) $$\begin{array}{c} \sum_{k=1}^{k}\left(\text{ykIn}\left(\text{pk}\right)+\left(1-\text{yk}\right)\text{In}\left(1-\text{pk}\right)\right), \end{array}$$

(36) $$\begin{array}{c} L=\prod_{k:yk=1}\left(\text{pk}\right)\prod_{k:yk=0}\left(1-\text{pk}\right). \end{array}$$

#### 3.3.6. Random Forest

A random forest is a meta estimator explained in equations (37) and (38), that uses averaging to improve prediction accuracy while minimizing overfitting. The subsample size is determined by the max-samples option, and each tree uses the entire dataset.

(37) $$\begin{array}{c} \hat{f}=\frac{1}{B}\sum_{B=1}^{B}\text{fb}\left(x^{\prime }\right), \end{array}$$

(38) $$\begin{array}{c} \sigma =\sqrt{\frac{\sum_{b=1}^{B}{\left(\text{fb}\left(x^{\prime }\right)\hat{f}\right)}^{2}}{B-1}}. \end{array}$$

#### 3.3.7. Stochastic Gradient Descent (SGD)

SGD has received significant attention, despite its long history in machine learning applications. Convex loss faced in SVM and LR is addressed by SGD. This technique (SGD) provides a quick and easy technique to fit linear classifiers and regressions in the context of large-scale learning. Equations (39)–(41) explain the SGD technique to provide a quick and best-fit machine learning classifier.

(39) $$\begin{array}{c} w=w-\eta \nabla Q\left(w\right), \end{array}$$

(40) $$\begin{array}{c} w-\frac{\eta }{n}\sum_{i=1}^{n}\nabla Q_{i}\left(w\right), \end{array}$$

(41) $$\begin{array}{c} W=w-\eta \nabla Q_{i}\left(w\right). \end{array}$$

#### 3.3.8. Performance Matrix

Several performance matrices are explained in equations (42)–(46), including accuracy, recall, precision, F1-score, and Matthews correlation coefficient (MCC). These evaluation parameters are used to check the performance of our proposed approach with other algorithms.

(42) $$\begin{array}{c} \text{Accuracy}=\frac{\text{Tp}+\text{TN}}{\text{TP}+\text{TN}+\text{FP}+\text{FN}}*100, \end{array}$$

(43) $$\begin{array}{c} \text{Precision}=\frac{\text{Tp}}{\text{TP}+\text{FP}}*100, \end{array}$$

(44) $$\begin{array}{c} \text{Recall}=\frac{\text{Tp}}{\text{TP}+\text{FN}}*100, \end{array}$$

(45) $$\begin{array}{c} F1-\text{score}=2*\frac{\text{Precision}*\text{Recall}}{\text{Precision}+\text{Recall}}, \end{array}$$

(46) $$\begin{array}{c} \text{MCC}=\frac{\text{Tp}*\text{TN}-\text{FP}*\text{FN}}{\left(\text{TP}+\text{FP}\right)\left(\text{TP}+\text{FN}\right)\left(\text{TN}+\text{FP}\right)\left(\text{TN}+\text{FN}\right)}*100, \end{array}$$

(47) $$\begin{array}{c} \text{Computational Time}=\text{software Time}*\text{Number of Features}*\text{Clock rate.} \end{array}$$

## 4. Result and Discussion

This section of the study provides various classification models and their statistical analysis. In the first phase, we compare the performance of LR, KNN, SGD, RF SVM, NB, and DT on the Cleveland heart disease dataset. In the second phase, we have employed LASSO, ANOVA, MultiSURF, variance threshold, and mutual information to pick relevant features. To evaluate the performance classifiers, all features were normalized and standardized before being supplied to classifiers.

The features of the entire dataset were tested on selected machine learning classifiers in this experiment, where 7 : 3 ratio data is allocated for training (70%) and testing (30%).

**Figure 2:**
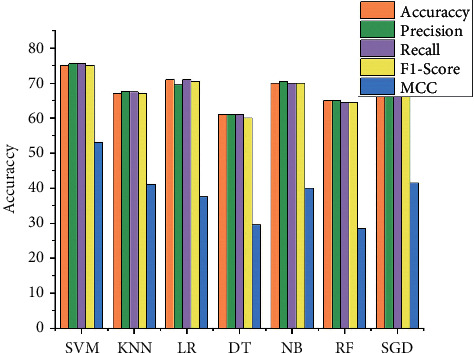
Result of the classifier with full feature.

In Table 2 and Figure 2, the SVM shows a good performance with 75% accuracy, 75.5% precision, 75.5% recall, 75% F1-score, 53% MMC, and 10.4 seconds time complexity. Different K values are tested for the KNN classifier, and the best performance among all round is; 67% accuracy, 67.6% precision, 67.5% recall, F1-score 67%, MCC 41%, and time complexity of 16.7 second. The LR classifier achieved 71% accuracy, 69.5% precision, 71% recall, 70.5% F1-score, MCC 37.5%, and time complexity is 12.2 second. The DT classifier achieved 61% accuracy, 61% precision, 61% recall, 60% F1-score, MCC 29.5%, and time complexity is 19.9 second. The NB classifier achieved 70% accuracy, 70.5% precision, 70% recall, 70% F1-score, MCC 40%, and time complexity is 24.7 second. The RF classifier achieved 65% accuracy, 65% precision, 64.5% recall, 64.5% F1-score, MCC 28.5%, and time complexity is 17.1 second. The SGD classifier achieved 69% accuracy, 69% precision, 69% recall, 68.5% F1-score, MCC 41.5%, and time complexity is 14.4 second.

**Figure 3:**
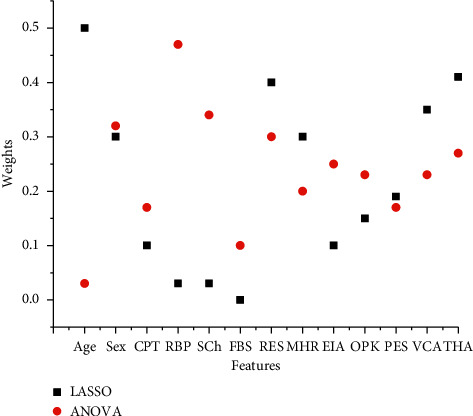
Feature selected with LASSO and ANOVA.

Based on their weight, LASSO and ANOVA select different features from the complete dataset. LASSO is used to select the five most important features namely SEX, RES, MHR, VCA, and THA. ANOVA select features, e.g., SEX, RBP, SCH, RES, and THA, as can be seen in Table 3 and Figure 3. We analyzed classifiers on a variety of chosen features and performances are very efficient.

**Figure 4:**
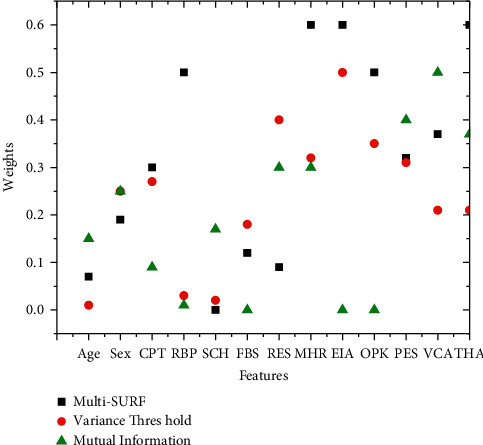
Selected feature with Multi-SURF, variance threshold, and mutual information.

The five most relevant features are selected and to be utilized in the second group of feature selection, namely MultiSURF, variance threshold, and mutual information, as shown in Table 3 and Figure 4. Multi-SURF selects RBP, MHR, EIA, OPK, and THA features from the dataset. RES, MHR, EIA, OPK, and PES features are the most prominent features for variance threshold. Moreover, RES, MHR, PES, VCA, and THA are chosen by mutual information select features which is the final and most essential feature selection algorithm.

**Figure 5:**
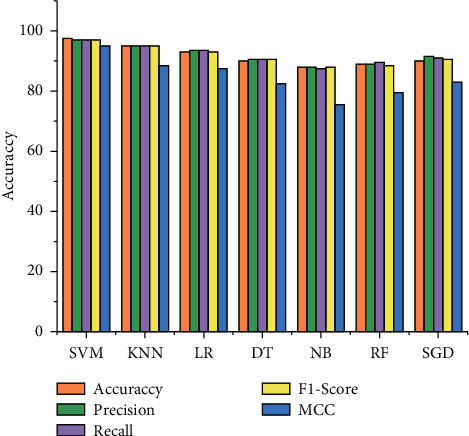
Result of the classifier with selected features.

As demonstrated in Figures 3 and 4, after features selection, the five most important features are tested on different machine learning classifiers, with a 7 : 3 ratio set for the training (70%) and testing (30%). In Table 4 and Figure 5, SVM shows a good performance by using a confusion matrix with 97.5% accuracy, 97% precision, 97% recall, 97% F1-score, 95% MMC, and 4.4 seconds time complexity. Different K values are applied for the KNN classifier and best among them are 95% accuracy, 95% precision, 95% recall, F1-score 95%, 88.5% MCC, and 7.3 seconds time complexity. The LR classifier achieved 93% accuracy, 93.5% precision, 93.5% recall, 93% F1-score, 87.5% MCC, and 8 seconds time complexity. The DT classifier has achieved 90% accuracy, 90.5% precision, 90.5% recall, 90.5% F1-score, 82.5% MCC, and 11 seconds time complexity. The NB classifier achieved 88% accuracy, 88% precision, 87.5% recall, 88% F1-score, 75.5% MCC, and 13.9 seconds time complexity. The RF classifier achieved 89% accuracy, 89% precision, 89.5% recall, 88.5% F1-score, 79.5% MCC, and 10 seconds time complexity. The SGD classifier achieved 90% accuracy, 91.5% precision, 91% recall, 90.5% F1-score, 83% MCC, and 12 seconds time complexity.

**Figure 6:**
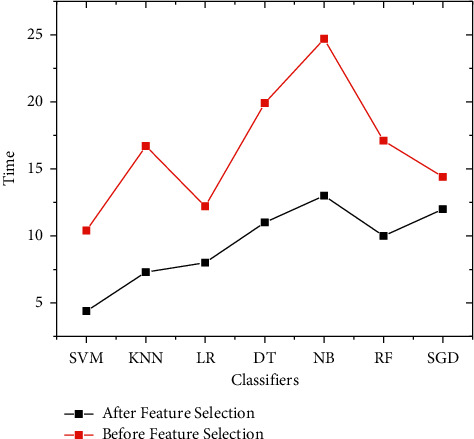
Time complexity with full features and selected features.

Figure 6 depicts the classifier parameters for overall features and five main characteristics to demonstrate time complexity of each classifier. The SVM algorithm has 4.4 seconds for selected features and 10.4 seconds for all other features in the dataset. KNN has 7.3 and 16.7 seconds, respectively. The LR algorithm has 8 and 12.2 seconds with and without features, the DT algorithm has 11 and 19.9 seconds, and the NB algorithm has 13.9 and 24.7 seconds. RF processing time for classifying the dataset is 10 and 17.1 seconds, and lastly, SGD has 12 and 14.4 seconds, respectively.

Table 5 illustrates an increase in SVM classification accuracy from 75% to 97.5% on minimized features. Similarly, the accuracy of KNN improved from 67% to 95% with reduced features, LR increased from 71% to 91%, DT increased from 61% to 90%, NB increased from 70% to 88%, RF increased from 65% to 89%, and SGD increased from 69% to 90%. As a result, the feature selection algorithms select significant features that boost the performance of the classifier and reduce execution time to effectively diagnose heart disease prediction.

### 4.1. Comparative Analysis

We employed several feature selection and machine learning approaches in the classification phase. The results demonstrated that our suggested methods produce efficient outcomes in terms of all performance matrices with minimum computational time. In the end, based on statistical data, we conclude that our proposed approach has improved the overall performance of algorithms as can be seen in Table 6.

## 5. Conclusion

This research study proposed a machine-learning-based cardiac disease classification system. Decision tree (DT), stochastic gradient descent (SGD), K-nearest neighbor (KNN), naive Bayes (NB), random forest (RF), logistics regression (LR), and support vector machine (SVM) were used to classify the Cleveland heart disease dataset collected from Cleveland hospitals. The novelty of this proposed work is the development of a diagnosis system for heart disease patients. Feature selection algorithms such as LASSO, ANOVA, MultiSURF, variance threshold, and mutual information are utilized before supplying data for the training and test phase, main motivation behind this approach is to improve the response time of each algorithm. Performance evaluation matrices, e.g., accuracy, precision, recall, F1-score, and MMC, were used to compare the different classifier performances. In addition, the proposed approach is evaluated on a 5-feature algorithm with 7 classifiers and 5 performance evaluation metrics and have shown efficient performance (refer to section 4). A machine learning classification model is used in this study. SVM, KNN, and LR models all perform well with specific features and can improve classification accuracy while also reducing the overall processing time. The findings are consistent with earlier research. In the future, we will apply federated learning and blockchain algorithms to generate an effective and efficient diagnosing system.

## Figures and Tables

**Algorithm 1:**
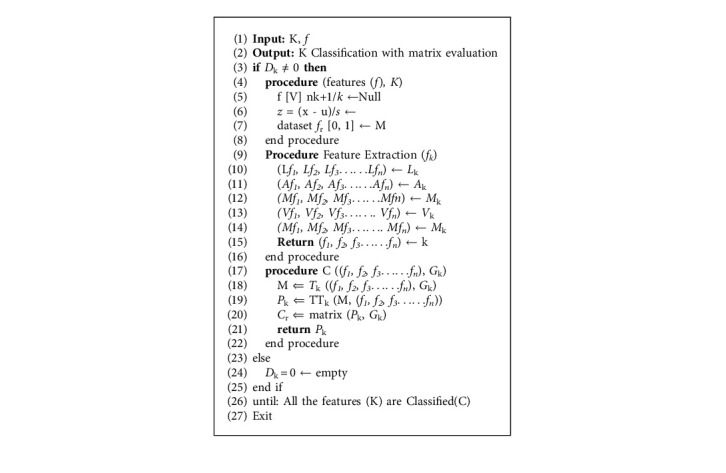
Heart disease classification; take input features, preprocessing, feature selection, and classification.

**Table 1:** Previous literature review.

Reference	Heart disease type	Application	ML algorithm	Approach	Evaluations (%)	Data
[23]	Coronary disease	Classification	CA, BA	Undersampling	71.1	425 patients data
[24]	General heart disease	Classification	MLP	Undersampling	80	Cleveland dataset
[25]	General heart disease	Classification	ANN	Sampling	84	Cleveland dataset
[26]	General heart disease	Three-phase system for the prediction	ANN	Data sampling	85	Uci
[5]	Heart disease	Ensemble-based predictive model	ANN	Undersampling	91	Cleveland heart disease
[27]	Coronary Heart disease	Adaptive fuzzy ensemble	GA, MS-pso	Feature selection	92.31	Public dataset
[28]	Coronary artery disease	Classification	SVM, NB	Feature selection	96	Z-Alizadeh sani dataset
[29]	Cardiac disease	Classification	SVM, DT, KNN, etc.	Focal loss	86	Cleveland heart disease
[30]	Cardiac arrest	Scoring system classification	SVM	Undersampling	78.8	1386 records
[31]	Heart disease (general)	Detection	NB, SMO	Features selection	83	Cleveland dataset
[32]	Coronary heart disease	Predication	SVM, KNN, etc.	SMOTE	72	African heart disease data
[33]	Arrhythmia	Diagnosing	SVM, KNN, DT, RF	SMOTE	92	MIT-BIH
[34]	Heart arrhythmia	Detection	XGBoost classifier	Undersampling	87	Biobank UK dataset
[35]	Chronic heart failure (HF)	Incremental and boosting features value	DT, RF, SVM, KNN, LMT	Undersampling	89	487 patient data
[36]	Cardiovascular diseases	Classification	RF, DT	SMOTE	91	4270 patients data
[37]	Heart disease (general)	Features method	Lda, KNN, SVM, RF	Sampling	84	UCI dataset
[38]	Heart arrhythmia	Classification	Marine predators algorithm, SGD, CNN	Sampling	99.47	MIT-BIH arrhythmia, European, INCART
[39]	Heart arrhythmia	Classification	Marine predators algorithm, DNN, CNN	Sampling	99	MIT-BIH, EDB, and INCART
[38]	Heart arrhythmia	Classification	Manta ray foraging optimization, SVM, LBP, HOS	Sampling	98.26	MIT-BIH arrhythmia

**Table 2:** Classifier performance before feature selection.

Classifier		Accuracy (%)	Precision (%)	Recall (%)	F1-score (%)	MCC (%)	Time complexity (sec)
SVM	1	75	80	73	76	51	10.4
	0	75	71	78	74	55	
	Overall	75	75.5	75.5	75	53	
KNN	1	67	64	69	66	35	16.7
	0	67	71	66	68	47	
	Overall	67	67.6	67.5	67	41	
LR	1	71	76	69	72	42	12.2
	0	71	63	73	69	33	
	Overall	71	69.5	71	70.5	37.5	
DT	1	61	56	62	58	22	19.9
	0	61	66	60	62	37	
	Overall	61	61	61	60	29.5	
NB	1	70	75	68	71	41	24.7
	0	70	66	72	69	39	
	Overall	70	70.5	70	70	40	
RF	1	65	68	64	66	30	17.1
	0	65	62	65	63	27	
	Overall	65	65	64.5	64.5	28.5	
SGD	1	69	76	66	71	39	14.4
	0	69	62	72	66	44	
	Overall	69	69	69	68.5	41.5	

**Table 3:** Selected feature rank.

Number	Algorithm		Feature name	Feature code	Rank
1	LASSO	1	Gender	SEX	0.3
		2	Resting electrocardiography	RES	0.4
		3	Maximum heart rate	MHR	0.3
		4	Number of major vessels	VCA	0.35
		5	Thallium scan	THA	0.41
2	ANOVA	1	Gender	SEX	0.32
		2	Level of BP	RBP	0.47
		3	Serum cholesterol	SCH	0.34
		4	Resting electrocardiography	RES	0.3
		5	Thallium scan	THA	0.27
	Multi SURF	1	Level of BP	RBP	0.5
		2	Maximum heart rate	MHR	0.6
		3	Exercise-induced angina	EIA	0.6
		4	Old peak	OPK	0.5
		5	Thallium scan	THA	0.6
4	Variance threshold	1	Resting electrocardiography	RES	0.4
		2	Maximum heart rate	MHR	0.32
		3	Exercise-induced angina	EIA	0.5
		4	Old peak	OPK	0.35
		5	Slope of the peak exercise	PES	0.31
5	Mutual information	1	Resting electrocardiography	RES	0.3
		2	Maximum heart rate	MHR	0.3
		3	Slope of the peak exercise	PES	0.4
		4	Number of major vessels	VCA	0.5
		5	Thallium scan	THA	0.37

**Table 4:** Selected feature result.

Classifier		Accuracy (%)	Precision (%)	Recall (%)	F1-score (%)	MCC (%)	Time complexity (sec)
SVM	1	97	96	98	97	94	4.4
	0	98	98	96	97	96	
	Overall	97.5	97	97	97	95	
KNN	1	95	92	98	95	90	7.3
	0	95	98	92	95	87	
	Overall	95	95	95	95	88.5	
LR	1	93	93	94	93	87	8
	0	93	94	93	93	88	
	Overall	93	93.5	93.5	93	87.5	
DT	1	90	90	93	91	81	11
	0	90	91	88	90	84	
	Overall	90	90.5	90.5	90.5	82.5	
NB	1	88	90	86	88	76	13.9
	0	88	86	89	88	75	
	Overall	88	88	87.5	88	75.5	
RF	1	89	92	87	89	78	10
	0	89	86	92	88	81	
	Overall	89	89	89.5	88.5	79.5	
SGD	1	90	85	95	90	82	12
	0	90	96	87	91	84	
	Overall	90	91.5	91	90.5	83	

**Table 5:** Improved accuracy result.

Classifier	Before feature selection (%)	After feature selection (%)
SVM	75	97.5
KNN	67	95
LR	71	93
DT	61	90
NB	70	88
RF	65	89
SGD	69	90

**Table 6:** Comparative analysis.

Matric Classifier	Proposed work	Previous work
	Accuracy	Accuracy
SVM	97.5%	88%
KNN	95%	81%
LR	91%	89%
DT	90%	83%
NB	88%	83%
RF	89%	66%
SGD	90%	N/A

## Data Availability

The data used to support the ﬁndings of the study are included in the article https://www.kaggle.com/datasets/aavigan/cleveland-clinic-heart-disease-dataset.
